# Characteristics of Disease-Specific and Generic Diagnostic Pitfalls: A Qualitative Study

**DOI:** 10.1001/jamanetworkopen.2021.44531

**Published:** 2022-01-21

**Authors:** Gordon D. Schiff, Mayya Volodarskaya, Elise Ruan, Andrea Lim, Adam Wright, Hardeep Singh, Harry Reyes Nieva

**Affiliations:** 1 Division of General Internal Medicine and Primary Care, Brigham and Women’s Hospital, Boston, Massachusetts; 2 Center for Patient Safety Research and Practice, Brigham and Women’s Hospital, Boston, Massachusetts; 3 Center for Primary Care, Harvard Medical School, Boston, Massachusetts; 4 Department of Surgery, Rush University Medical Center, Chicago, Illinois; 5 Department of Medicine, Montefiore Medical Center, Bronx, New York; 6 Department of Internal Medicine, Kaiser Permanente, San Francisco, California; 7 Department of Biomedical Informatics, Vanderbilt University, Nashville, Tennessee; 8 Center for Innovations in Quality, Effectiveness and Safety, Michael E. DeBakey Veterans Affairs Medical Center and Baylor College of Medicine, Houston, Texas; 9 Department of Medicine, Harvard Medical School, Boston, Massachusetts; 10 Department of Biomedical Informatics, Columbia University, New York, New York

## Abstract

**Importance:**

Progress in understanding and preventing diagnostic errors has been modest. New approaches are needed to help clinicians anticipate and prevent such errors. Delineating recurring diagnostic pitfalls holds potential for conceptual and practical ways for improvement.

**Objectives:**

To develop the construct and collect examples of “diagnostic pitfalls,” defined as clinical situations and scenarios vulnerable to errors that may lead to missed, delayed, or wrong diagnoses.

**Design, Setting, and Participants:**

This qualitative study used data from January 1, 2004, to December 31, 2016, from retrospective analysis of diagnosis-related patient safety incident reports, closed malpractice claims, and ambulatory morbidity and mortality conferences, as well as specialty focus groups. Data analyses were conducted between January 1, 2017, and December 31, 2019.

**Main Outcomes and Measures:**

From each data source, potential diagnostic error cases were identified, and the following information was extracted: erroneous and correct diagnoses, presenting signs and symptoms, and areas of breakdowns in the diagnostic process (using Diagnosis Error Evaluation and Research and Reliable Diagnosis Challenges taxonomies). From this compilation, examples were collected of disease-specific pitfalls; this list was used to conduct a qualitative analysis of emerging themes to derive a generic taxonomy of diagnostic pitfalls.

**Results:**

A total of 836 relevant cases were identified among 4325 patient safety incident reports, 403 closed malpractice claims, 24 ambulatory morbidity and mortality conferences, and 355 focus groups responses. From these, 661 disease-specific diagnostic pitfalls were identified. A qualitative review of these disease-specific pitfalls identified 21 generic diagnostic pitfalls categories, which included mistaking one disease for another disease (eg, aortic dissection is misdiagnosed as acute myocardial infarction), failure to appreciate test result limitations, and atypical disease presentations.

**Conclusions and Relevance:**

Recurring types of pitfalls were identified and collected from diagnostic error cases. Clinicians could benefit from knowledge of both disease-specific and generic cross-cutting pitfalls. Study findings can potentially inform educational and quality improvement efforts to anticipate and prevent future errors.

## Key Points

**Question:**

Are there similarities among clinical situations associated with diagnostic errors?

**Findings:**

This qualitative study identified 836 relevant cases among 4325 patient safety incident reports, 403 closed malpractice claims, 24 morbidity and mortality reports, and 355 focus group responses. From these, 661 disease-specific and 21 generic diagnostic “pitfalls” were identified.

**Meaning:**

Diagnostic pitfalls represent a potentially useful construct that bridges cognitive and systems diagnosis error approaches because they can delineate and demonstrate recurrent patterns of diagnostic error.

## Teaser

This qualitative study develops the construct and collects examples of diagnostic pitfalls, defined as clinical situations and scenarios vulnerable to errors that may lead to missed, delayed, or wrong diagnoses.

## Introduction

Diagnostic errors are the leading type of medical error reported by patients^1^ and a leading cause for malpractice claims.^2,3,4^ A study of malpractice claims in primary care in Massachusetts found that more than 70% of primary care claims reside in the diagnostic safety realm, and patients rank diagnostic errors as the leading type of medical error that they have experienced.^5^ In 2015, the National Academy of Medicine issued a report, *Improving Diagnosis in Health Care,* highlighting the importance and causes of diagnostic errors and making recommendations for preventing and mitigating such errors.^6^ However, despite increasing appreciation of diagnostic errors as a patient safety issue, progress in understanding and preventing diagnostic errors has been modest.^7^ Unlike medication errors, which have been more successfully reduced with system-level and information technology–based interventions, there are no comparable single technical or educational fixes for diagnostic errors.^8,9^

One central and recurring theme has centered around efforts to differentiate so-called cognitive errors from system errors.^6,10,11^ However, emerging evidence suggests that most diagnostic errors are multifactorial, with these cognitive and system factors often overlapping and interacting.^12,13^ We sought to develop a new approach—“diagnostic pitfalls”—that recognizes and bridges this overlap and that may potentially provide practical, disease-specific guidance to help clinicians and organizations consider, anticipate, identify, and mitigate what can go wrong in diagnosis.

The concept of diagnostic pitfalls is hardly a new notion. It was highlighted more than a century ago by Cabot in the article “Diagnostic Pitfalls Identified During a Study of Three Thousand Autopsies.”^14^ Nonetheless, during the ensuing decades, the term has been variably and inconsistently used in the medical literature but rarely invoked in current diagnosis research. In this study, we aim to review a comprehensive series of diagnostic error cases and identify both diagnosis-specific and generic cross-cutting issues that frequently occur as pitfalls.

We define diagnostic pitfalls as clinical situations and scenarios that are vulnerable to errors that may lead to missed, delayed, or wrong diagnoses. This practical construct embraces both cognitive issues (eg, knowledge gaps, heuristics, and biases) and system factors (eg, communication breakdowns, disease presentation factors, and test limitations) associated with diagnostic error. We used a multisource approach to collect diagnostic error cases to delineate potential pitfalls. This study had 3 aims: (1) to develop and refine the construct of diagnostic pitfalls, (2) to collect a list of disease-specific examples of diagnostic pitfalls, and (3) to analyze these disease-specific pitfalls to create a taxonomy of generic types of diagnostic pitfalls occurring in primary care practice. Knowledge of such potential diagnostic pitfalls could help clinicians and organizations design educational efforts and safety nets to anticipate, prevent, and/or mitigate such errors.

## Methods

### Study Design, Setting, and Participants

We analyzed data from physicians and adult patients in ambulatory practices and academic medical centers across Massachusetts between January 1, 2004, and December 31, 2016. All cases involved diagnostic errors and data originated from (1) closed malpractice claims, (2) patient safety incident reports, (3) ambulatory morbidity and mortality conferences, or (4) specialty focus groups. For all data sources, we reviewed each case and abstracted key variables of interest. After aggregating abstracted data, we calculated summary statistics, conducted a qualitative thematic analysis of disease-specific challenges, and then iteratively derived generic diagnostic pitfalls. This study followed the Standards for Reporting Qualitative Research (SRQR) reporting guideline and was approved by the Mass General Brigham institutional review board.

### Data Collection

For all data sources, a trained research assistant reviewed each clinical case in consultation with a general internist (G.D.S.) with expertise in diagnostic error. We abstracted the same key data elements from each source and recorded them in an Access (Microsoft Corp) database using a single standardized form with preset, drop-down fields and a free-text box to briefly summarize each case. Variables of interest included data source (eg, closed malpractice claim), erroneous (ie, initial) diagnosis, correct diagnosis, and presenting signs and symptoms. We also classified breakdowns in the diagnostic process using 2 complementary taxonomies, Diagnosis Error Evaluation and Research (DEER)^12,15^ (eTable 1 in the Supplement) and Reliable Diagnosis Challenges (RDC)^16^ (eTable 2 in the Supplement), assigning up to 3 DEER codes and 3 RDC codes for each case. DEER identifies what went wrong and situates where the failure occurred in the diagnostic process, whereas RDC identifies the general challenges complicating the diagnostic process and the potential reasons a mistake occurred.

### Data Sources

We pooled data from all 4 sources and reviewed each case scenario for eligibility for qualitative coding. Eligibility was broadly defined as cases previously coded by the 2 malpractice insurers as diagnosis related as well as cases in the institutional databases or collected from specialty focus groups that could be considered diagnostic errors or delays.

#### Patient Safety Incident Reports

We extracted 11 years of diagnosis-related patient safety incident reports (2004-2014) from a large academic medical center in eastern Massachusetts using RL Solutions, a commercial incident management solution. The Patient Safety/Risk Management department reviews every report on a daily basis and fills in fields that were left blank or required follow-up to clarify additional information.

#### Closed Malpractice Claims

We reviewed 5 years of closed malpractice claims involving adult primary care ambulatory clinics (ie, internal and family medicine) from 2 malpractice insurers, Controlled Risk Insurance Company (CRICO) and Coverys, which, when combined, insure more than 85% of all clinicians in Massachusetts. All cases were coded by insurers by professional medical coders with nursing experience. We restricted our review to all closed claims assigned a diagnostic error tag with an initial claim date between 2010 and 2014.

#### Ambulatory Morbidity and Mortality Rounds

We collated information on all available primary care morbidity and mortality conferences at our academic medical center over the 11-year period between 2004 and 2014. We restricted cases to those involving diagnostic errors in the ambulatory setting of care. To obtain information, we solicited materials from departmental internal online repositories.

#### Physician Focus Groups

Between March 2015 and December 2016, we put together 6 one-hour focus groups with attending physicians and clinical fellows during regularly scheduled division meetings or educational conferences for the following specialties: (1) neurology, (2) gastroenterology, (3) dermatology, (4) pulmonary and critical care medicine, (5) rheumatology, and (6) oral medicine and dentistry. We targeted these as examples of medical specialties that we postulated were in a position to observe diagnostic errors and delays that occurred upstream in the course of patients’ primary care. After obtaining oral informed consent, we oriented the specialists to the problem of diagnostic errors and the concept of diagnostic pitfalls. Participants received a paper form to solicit up to 3 examples of diagnostic pitfalls, asking “What kind of disease-specific diagnostic pitfalls, errors, or mistakes do you most commonly observe primary care physicians make?” Participants were allowed 15 minutes to provide written examples; we then engaged them in a 30-minute discussion to discuss their examples. The written responses were collected, and the sessions were digitally recorded and transcribed.

### Statistical Analysis

Data analyses were performed between January 1, 2017, and December 31, 2019. We aggregated data abstracted from all sources and calculated descriptive statistics based on DEER and RDC coding. We also computed the most frequently missed or delayed diagnoses by disease and system and noted the most frequent signs or symptoms mentioned. Next, we performed a preliminary review of all cases to collate disease-specific examples that met our preestablished definition of diagnostic pitfalls. We then conducted an iterative thematic analysis of these disease-specific pitfalls to derive generic diagnostic pitfalls. We started by familiarizing ourselves with the data, using DEER and RDC taxonomies as preliminary codes to provide structure and describe the content, then searched for patterns or themes across cases, reviewed these themes until reaching saturation, and, finally, named and defined those broader themes. Analysis occurred iteratively to allow for regular, real-time identification and interpretation of recurrent patterns, organizing of themes, verification of accuracy and consistency of findings, and adjudication of any disagreement.^17^ We assigned each case up to 3 generic diagnostic pitfalls based on this final list.

## Results

**Figure 1.**
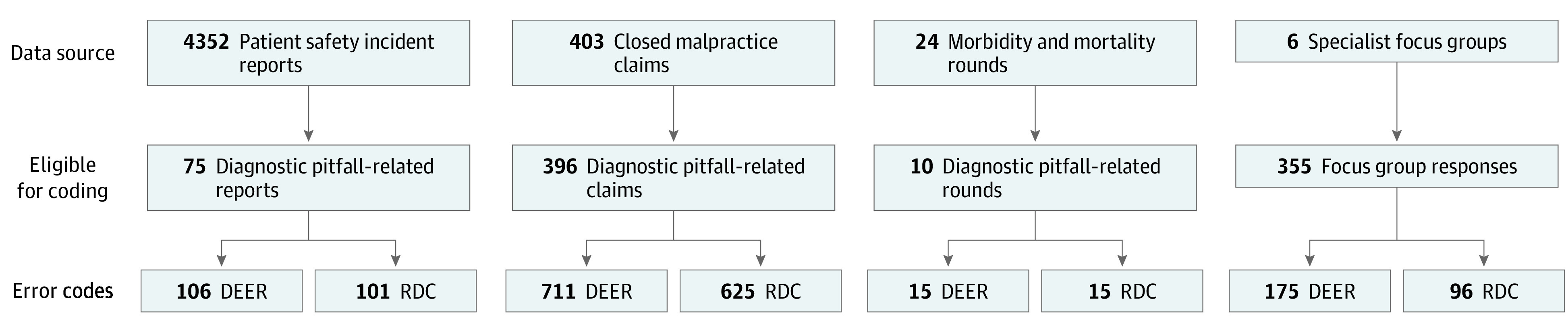
Diagnostic Errors Identified and Coded Using Diagnosis Error Evaluation and Research (DEER) and Reliable Diagnosis Challenges (RDC) Taxonomies

Our data sources included 4352 patient safety incident reports, 403 closed malpractice claims, 24 ambulatory morbidity and mortality rounds, and 355 focus group responses collected over 6 sessions among physicians from 6 specialties (Figure 1). Focus groups ranged in size from 8 to 25 specialist physician participants. We reviewed each case for eligibility and identified 836 relevant diagnostic error cases comprising 75 of 4352 incident reports (2%), 396 of 403 closed claims (98%), 10 of 24 morbidity and mortality conferences (42%), and all 355 focus group responses.

Table 1 lists the top 10 most commonly missed or delayed diagnoses by condition and system (836 cases). The most frequent diagnoses were colorectal (38 [5%]), lung (36 [4%]), breast (20 [2%]), prostate (18 [2%]), and bladder (10 [1%]) cancers; myocardial infarction (20 [2%]); stroke (15 [2%]); sepsis (13 [2%]); pulmonary embolism (9 [1%]); and brain hemorrhage (8 [1%]). The most common diagnoses by system were oncology, neurology, and cardiology diagnoses (reflecting in large part the case contributions from the focus groups). Pain (abdominal, general, and chest), emesis, fever, headache, and altered mental status were the most frequent presenting signs and symptoms.

**Table 1.** Top 10 Missed or Delayed Diagnoses by Condition and System^a^

Diagnosis	No. (%) (N = 836)
By condition	
1. Colorectal cancer	38 (5)
2. Lung cancer	36 (4)
3. Breast cancer	20 (2)
4. Myocardial infarction	20 (2)
5. Prostate cancer	18 (2)
6. Stroke	15 (2)
7. Sepsis	13 (2)
8. Bladder cancer	10 (1)
9. Pulmonary embolism	9 (1)
10. Brain hemorrhage	8 (1)
By system	
1. Oncology	225 (27)
2. Neurology	89 (11)
3. Cardiology	50 (6)
4. Infectious diseases	46 (6)
5. Other	40 (5)
6. Dermatology	37 (4)
7. Gastroenterology	35 (4)
8. Pulmonology	33 (4)
9. Rheumatology	29 (3)
10. Orthopedics	16 (2)

^a^ Derived from 836 relevant cases among 4325 patient safety incident reports, 403 closed malpractice claims, 24 morbidity and mortality reports, and 355 focus groups responses.

**Figure 2.**
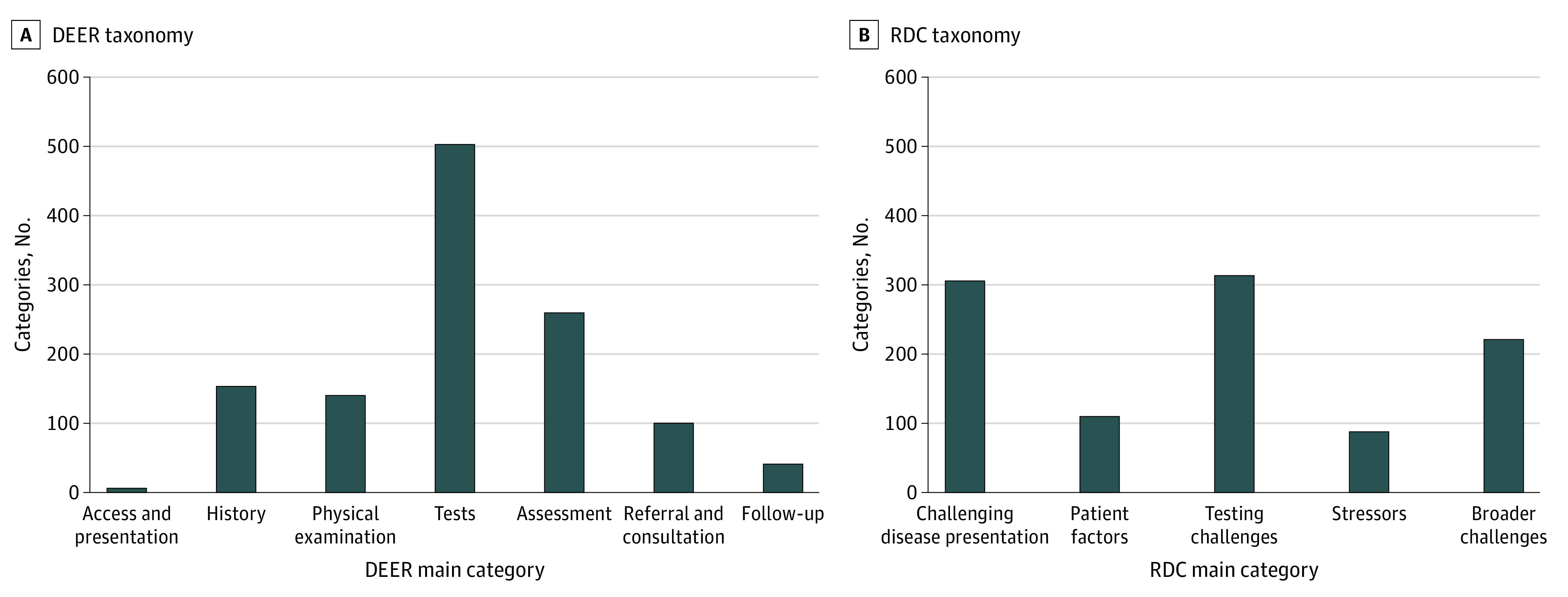
Classification by Diagnosis Error Evaluation and Research (DEER) and Reliable Diagnosis Challenges (RDC) Taxonomies

Based on DEER taxonomy coding of the diagnostic process from access or presentation through follow-up (Figure 2A), 63% of 1208 errors were “localized” to the testing (n = 503) and assessment (n = 260) phases. The most common DEER subcategories were failure in ordering the needed test (n = 214), failure to consider the correct diagnosis (n = 144), failure to or delay in follow-up of (abnormal) test results (n = 107), failure in weighing a critical piece of history data (n = 94), and failure to order or delay in ordering a referral (n = 83).

Coding using the RDC taxonomy (Figure 2B) revealed that 59% of 1041 errors were associated with testing challenges (n = 314) and challenging disease presentation (n = 305). The most frequent RDC subcategories assigned were test follow-up issues (n = 131), challenges in recognition of acuity or severity of illness (n = 82), test performance or interpretation (n = 69), masking or mimicking diagnosis (n = 64), and failure to diagnose the underlying cause (n = 62).

Most specialists were able to offer a number of specific examples of diagnosis failures that they considered to represent recurring pitfalls. These examples generally clustered into (1) more serious specialty diagnoses that were missed or delayed as a result of misdiagnosis upstream by the primary care physician; (2) instances of misdiagnosis or overdiagnosis in which patients were labeled as having a specialty diagnosis (eg, multiple sclerosis) that did not meet diagnostic criteria and/or where an alternate diagnosis (usually more benign, nonspecific, or psychiatric in nature) was more likely; and (3) generic pitfalls in clinical examination or testing relevant to that specialty (see eTable 3 in the Supplement for diagnostic pitfalls associated with neurologic conditions).

From this pool of eligible cases, we identified 661 disease-specific pitfalls. Illustrative disease-specific pitfalls included (1) misreading a lung mass as pneumonia on chest radiograph; (2) ordering screening, instead of diagnostic, mammogram in evaluation of breast lump (see eTable 4 in the Supplement for additional breast cancer examples); (3) attributing intermittent hematuria to urinary tract infection despite negative urine cultures, thereby missing bladder cancer; (4) misinterpreting facial flushing as rosacea, delaying diagnosis of carcinoid syndrome; and (5) underweighing the possibility of transient ischemic attack in a patient with bilateral neurologic symptoms.

Although our primary aim was to collect disease-specific pitfalls, we also sought to understand the different types of recurring cross-cutting generic issues that characterized these distinct pitfalls. Based on the patterns and themes noted among disease-specific pitfalls, we derived 21 generic diagnostic pitfalls (Table 2) and grouped them using DEER categories. Prominent examples of these generic pitfalls include one disease misdiagnosed as another disease (eg, colon cancer erroneously diagnosed as hemorrhoids or celiac disease; bipolar disorder labeled or misdiagnosed as depression), failure to appreciate the limitations of a test or examination (eg, patient with breast lump and negative mammogram or ultrasonography result), atypical presentation (eg, Addison disease presenting with weight loss, cognitive difficulties, and fatigue), presuming chronic disease accounts for new symptoms (eg, attributing weight loss and cough to a patient’s chronic obstructive pulmonary disease, leading to delayed lung cancer diagnosis), and failure to monitor evolving symptoms (eg, normal results from cranial imagining shortly after head injury but chronic subdural hematoma later developed).

**Table 2.** Generic Diagnostic Pitfall Categories^a^

Generic diagnostic pitfall	Illustrative examples
**Diagnosis and assessment**	
One disease misdiagnosed or confused with another disease	Nasopharyngeal carcinoma misdiagnosed as recurrent sinusitis, CHF misdiagnosed as asthma, GERD misdiagnosed as asthma, burning mouth syndrome misdiagnosed as psychogenic pain, stasis dermatitis misdiagnosed as cellulitis
Misled by atypical presentation	Subarachnoid aneurysms presenting as isolated eye pain
Rare diagnosis: failure to consider or know	Ewing sarcoma in patient with bone pain and tiredness
Chronic disease presumed to account for new symptoms (especially in patients with medically complex conditions)	Septic joint misattributed to patient’s chronic gouty arthritis, Hodgkin lymphoma lymphadenopathy ascribed to patient’s chronic sarcoidosis
Counterdiagnosis cues overlooked (eg, red flags, things that do not fit are not recognized)	Lung cancer: infiltrate seen on chest radiograph with failure to respond to repeated antibiotic courses not recognized
Drug or environmental factor overlooked as cause of symptoms or as cause of disease progression	Bladder cancer in patient taking pioglitazone, which is known risk factor; tongue swelling as manifestation of angioedema from ACE inhibitors
No specific diagnosis is made at initial or subsequent encounters	Multiple cases
**History and physical**	
Nonspecific or vague symptom(s); hard-to-pinpoint diagnosis	Nonspecific dizziness, abdominal pain, weakness on initial presentation as symptoms of various more serious diagnoses
Intermittent symptoms: overlooked because findings (eg, examination, laboratory test, ECG) negative when patient seen	Recurrent PSVT misdiagnosed as anxiety, vasovagal syncope misdiagnosed as seizure disorder
Failure to appreciate risk factor(s) (or those at risk) for a given disease	Cervical cancer in patient with in utero DES exposure
Failure to appreciate limitations of the physical examination	Appendicitis: presentation without fever or localizing abdominal examination
**Testing**	
Failure to appreciate limitations of diagnostic test result(s)	Breast lump with negative mammography result erroneously interpreted as ruling out cancer; thoracic spinal epidural abscess missed when lumbosacral MRI performed; positive ANA result: multiple cases misdiagnosed as SLE
Failure in follow-up of abnormal or critical result	Multiple cases: elevated PSA, positive troponin, anemia, lung nodule
**Communication**	
Communication failure with patient, including language barriers	Missing critical history items in Spanish-speaking patients
Failure around communication related to ordering of laboratory test or imaging tests	Failure to communicate with radiology when ordering mammography that patient has a palpable breast lump, leading to performance of screening rather than diagnostic mammography
Communication failure between clinicians (eg, PCP and specialist, ED and PCP)	Multiple handoff failures to follow up on incidental findings uncovered in ED by specialists (eg, pulmonary nodules found on results of spiral CT scan performed in ED to rule out PE)
**Follow-up**	
Failure to monitor, note, or respond to evolving, continuing, or persistent symptoms	Pancreatic cancer: progressing weight loss with failure to note or evaluate; TB: failure to monitor continuing fever not responding to antibiotics for community pneumonia
Inadequate follow-up visits or referrals, especially in the presence of diagnostic uncertainty	Multiple instances of no or delayed follow-up visits
**Other**	
Urgency of the clinical situation was not appreciated	Rapidly progressing spinal cord compression in cancer, spinal epidural abscess
Diagnostic findings were masked or misinterpreted owing to an intervention or drug (eg, empirical treatment with oral or topical corticosteroids, PPI, antibiotics, pain medications)	Gastric cancer diagnosis delay resulting from mistaken reassurance from relief from antacids; antipyretics falsely masking patient’s fever; prior antibiotic administration resulting in negative culture results
Problems with inappropriate referral or overreferral	

Abbreviations: ACE, angiotensin-converting enzyme; ANA, antinuclear antibody; CHF, congestive heart failure; CT, computed tomography; DES, diethylstilbestrol; ECG, electrocardiogram; ED, emergency department; GERD, gastroesophageal reflux disease; MRI, magnetic resonance imaging; PCP, primary care physician; PE, pulmonary embolism; PPI, proton pump inhibitor; PSA, prostate-specific antigen; PSVT, paroxysmal supraventricular tachycardia; SLE, systemic lupus erythematosus; TB, tuberculosis.

^a^ Derived from iterative thematic analysis of 836 relevant cases among 4325 patient safety incident reports, 403 closed malpractice claims, 24 morbidity and mortality reports, and 355 focus groups responses.

For the most frequent diagnoses (which were dominated by malpractice cases, given the larger sample of these cases), we illustrate the types of generic diagnostic pitfalls in eTable 5 in the Supplement. The relative frequency of the 5 leading types of generic pitfalls were failure to follow-up, not appreciating test limitations, mistaking one disease for another disease (eg, aortic dissection is misdiagnosed as acute myocardial infarction), failure to appreciate patient risk factors, and atypical disease presentation.

## Discussion

Using multiple sources, we identified a series of diagnostic error cases that we used to help characterize failures in the diagnostic process in primary care. We used these instances of opportunities to improve diagnoses to compile a list of disease-specific examples as well as create a generic taxonomy of the types of recurring scenarios and issues—vulnerabilities in the diagnostic process that we have termed *diagnostic pitfalls*. By exploring both traditional sources of diagnostic error cases, such as malpractice claims and organizational error reports, and more novel inputs, such as errors identified by specialists with patients referred by primary care physicians, we were able to compile a rich collection of cases to review for pitfalls.

We used 2 previously validated tools for classifying where in the diagnostic process (DEER) and why (RDC) these diagnoses were potentially challenging for clinicians.^15,16^ Similar to prior studies,^15^ we found that issues associated with diagnostic testing predominated (especially ordering [mainly failure to order], interpretation, and follow-up of abnormal results) and patient assessment (particularly in failure to consider a diagnosis, recognize disease severity or urgency, and recognize atypical disease presentation or one that mimicked or was masked by a competing diagnosis). As in earlier studies, many of these cases had overlapping and multifactorial issues, suggesting the need for a multifaceted approach to recognize and prevent such errors.^10,11,15^

To make diagnoses more reliable, we need to enhance the ability of clinicians and systems to anticipate what can go wrong and build strategies to minimize vulnerabilities associated with these pitfalls.^18^ Compiling a list of potential pitfalls represents an essential first step for additional interventions. For instance, in addition to educational interventions focused on clinicians anticipating these pitfalls, such pitfalls could inform the development of decision-support interventions that warn clinicians in real time to avoid these errors.

A key feature of high-reliability organizations is continual awareness and worry about what can go wrong.^19,20,21,22^ In applying lessons from a retrospective review of errors, the distillation of recurring pitfalls can help frontline clinicians anticipate these pitfalls and thereby recognize and prevent future errors.^23^ Diagnostic pitfalls can potentially fulfill such a role, warning of diagnosis-specific risks as well as providing a more generalized awareness of omnipresent vulnerabilities inherent in uncertain diagnoses (Box).^24^ Nevertheless, because education and warnings are lower in the hierarchy of effectiveness of improvement interventions, operationalizing systems to enhance situational awareness and building safety nets to minimize such errors are also a necessity.^25^

Box. **Potential Ways the “Pitfalls” Construct Can Be Useful**

**Facilitate efforts and approaches to reduce diagnostic errors**

More clinically oriented and engaging to clinicians than other diagnostic error approaches (cognitive biases, industrial CQI methods)
Lends itself to fewer defensive responses from clinicians—by showing recurring pitfalls, vulnerabilities, and errors others have made, clinicians feel less singled out when their own diagnoses are in error
Shining light on recurring pitfalls can provide motivation to stimulate clinicians, practices, and organizations to address them to prevent recurrences
Complements other heuristics’ didactics (eg, biases) by providing actual examples of recurring failures or vulnerabilities
Provides an interoperable framework for conversations with and across specialties regarding disease-specific diagnostic vulnerabilities

**Provide practical levers to catalyze change**

Provides an educational framework to communicate and impart experiences with misdiagnosis for particular symptoms or diagnoses for educating new or practicing clinicians
Inform new section of medical textbooks to supplement usual sections on epidemiology, pathophysiology, diagnosis, and treatment; this could involve creating a new pitfalls section listing common and important pitfalls to anticipate and avoid in diagnosis
Potential to facilitate electronic diagnostic decision support via context-aware warnings of lurking pitfalls (eg, easily confused diagnoses, test limitations, atypical presentations, and causes to consider); to avoid overalerting, the warnings could be in the background to be queried by clinicians (click Alt-P to activate) when pondering uncertain or dangerous clinical situations
Inform triggers to retrospectively search for past or potentially ongoing diagnostic errors in clinical databases (eg, search for women with breast lump referred for screening, rather than diagnostic, mammogram; search abnormal mammograms that are not followed up)
Ability to identify vulnerable situations to prioritize and improve process redesign to immunize systems and clinicians to prevent, detect, and mitigate errors (eg, design forcing functions to serve as “guard rails” against that pitfall)
Framework for collecting epidemiologic data on incidence, risk factors, and high-risk situations for disease-specific pitfalls
Patient tool to aid patients in reviewing list of pitfalls online to raise questions about potential pitfalls in their own diagnosis (eg, could my negative COVID-19 test result be a false-negative result?)

Abbreviation: CQI, continuous quality improvement.

For instance, beyond simply anticipating errors, it is critical to put in place mechanisms to prevent their occurrence or mitigate their harm. Our data identified multiple examples of well-known pitfalls, for example, misdiagnosing acute aortic dissection as acute myocardial infarction (a more common acute cardiac condition) or concluding that a woman who has a palpable breast lump and negative mammogram results has had breast cancer “ruled out” (eTable 4 in the Supplement). This findings suggest that awareness of pitfalls needs to be supplemented with ways to remind clinicians in real time and with design mechanisms to provide forcing functions to potentially avoid such pitfalls (eg, mammography protocols requiring women undergoing screening mammography to fill out a form inquiring whether they had or are being referred for a breast lump).

Identifying disease-specific failures offers many ways for making progress in understanding and preventing diagnostic errors (Box). It holds the potential for guidance for a more granular understanding of what went wrong in the diagnostic process, a need highlighted by the National Quality Forum report on improving diagnostic quality and safety.^26^ By focusing on specific clinical scenarios, this approach has the potential to better engage practicing physicians in ways that industrial-improvement language related to “systems redesign” may fail to speak to and spark their clinical imaginations. It also holds potential to better link work being done in the general diagnostic error realm with specialists and specialty societies and researchers, who are working in the silos of their respective diseases.^27,28,29^ Generic pitfalls provide a framework for bridging the gap across specific diseases. Specific pitfalls can also be operationally defined to inform the development and deployment of electronic triggers to retrospectively examine and understand their occurrence and an institution’s vulnerability to these types of missteps.^30^

### Strengths and Limitations

This study has some strengths. Although it uses a convenience sample, our approach integrated high-quality data sources rich in examples of diagnostic error and included a wide range of diseases, which resulted in a substantial, broad set of diagnostic errors for analyses. To support uniform data collection, we used the same structured abstraction form across all data sources.

Nonetheless, we recognize that our findings should also be considered in light of potential limitations. Our approach is prone to potential bias in representativeness of diagnoses owing to underreporting and selective reporting of patient safety incidents, malpractice claims filed, morbidity and mortality cases presented, and the specialist physicians who participated in our focus groups. Although data sources spanned multiple specialties, the focus groups were a convenience sample of 6 specialties, and all information sources, except for closed malpractice claims, originated from a single academic medical center. We also recognize that coding cases using DEER and RDC taxonomies may be subject to reviewer bias and experience, although these were previously validated tools with good reliability and all cases were discussed and secondarily reviewed by a diagnosis error expert internist. Given that our data sources represent a convenience sample, some from older retrospective data, we were unable to estimate the true current prevalence of these diagnostic pitfalls. Accordingly, we only report frequencies and emphasize the general principles and themes identified. Some of these examples possibly risk overinterpretation if potentially rare causes or diagnoses were oversampled, risking “overdiagnosis” types of errors if uncritically applied. Thus, the implications for clinical practice merit cautious and careful consideration to avoid excessive worry or overdiagnosis (eg, in many cases, breast lumps, headaches, or rectal bleeding do not herald cancer).^31^

## Conclusions

Disease-specific and generic diagnostic pitfalls represent a potentially useful construct that bridges the gap across disease, system, and cognitive factors associated with diagnostic errors in medicine. We were able to identify and classify such pitfalls using cross-sectional lenses of locally reported cases, regional malpractice claims, and specialty expert input. Pitfalls can help illustrate specific and recurrent types of errors for specific diagnosis and clinical situations, as well as illustrate crosscutting themes that can be applied more broadly across diseases. Distillation of diagnostic pitfalls offers a number of benefits for making progress in understanding and preventing diagnostic errors and in better understanding what went wrong, how frequently the error occurs, where in the diagnostic process it occurs and why it occurs, and for providing clinically rich examples to show frontline clinicians situations that are predisposed to errors.

## Notes

Supplement. **eTable 1.** Diagnosis Error Evaluation and Research (DEER) Taxonomy **eTable 2.** Reliable Diagnosis Challenges (RDC) Taxonomy **eTable 3.** Diagnostic Pitfalls Associated With Neurological Conditions **eTable 4.** Diagnostic Pitfalls Associated With Breast Cancer **eTable 5.** Generic Diagnostic Pitfalls Associated With Most Frequent Conditions
