# Supplementary material for: Characteristics of Disease-Specific and Generic Diagnostic Pitfalls: A Qualitative Study
Source: JAMA Netw Open. 2022 Jan 21;5(1):e2144531. doi: 10.1001/jamanetworkopen.2021.44531 (PMC8783262; doi:10.1001/jamanetworkopen.2021.44531)
Supplement: Supplement. — eTable 1. Diagnosis Error Evaluation and Research (DEER) Taxonomy eTable 2. Reliable Diagnosis Challenges (RDC) Taxonomy eTable 3. Diagnostic Pitfalls Associated With Neurological Conditions eTable 4. Diagnostic Pitfalls Associated With Breast Cancer eTable 5. Generic Diagnostic Pitfalls Associated With Most Frequent Conditions [file jamanetwopen-e2144531-s001.pdf]

Supplementary Online Content

Schiff GD, Volodarskaya M, Ruan E, et al. Characteristics of disease-specific and generic diagnostic pitfalls: a qualitative study. *JAMA Netw Open.* 2022;5(1):e2144531. doi:10.1001/jamanetworkopen.2021.44531

eTable 1. Diagnosis Error Evaluation and Research (DEER) Taxonomy

eTable 2. Reliable Diagnosis Challenges (RDC) Taxonomy

eTable 3. Diagnostic Pitfalls Associated With Neurological Conditions

eTable 4. Diagnostic Pitfalls Associated With Breast Cancer

eTable 5. Generic Diagnostic Pitfalls Associated With Most Frequent Conditions

This supplementary material has been provided by the authors to give readers additional information about their work.

© 2022 Schiff GD et al. *JAMA Network Open.*

eTable 1. Diagnosis Error Evaluation and Research (DEER) Taxonomy

Where in diagnostic process			*What went wrong*
1. Access/Presentation		A	Failure/delay in presentation
		B	Failure/denied care access
2. History		A	Failure/delay in eliciting critical piece of history data
		B	Inaccurate/misinterpretation
		C	Failure in weighing
		D	Failure/delay to follow-up
3. Physical Exam		A	Failure/delay in eliciting critical physical exam finding
		B	Inaccurate/misinterpreted
		C	Failure in weighing
		D	Failure/delay to follow-up
4. Tests (Lab/Radiology)			*Ordering*
		A	Failure/delay in ordering needed test(s)
		B	Failure/delay in performing ordered test(s)
		C	Error in test sequencing
		D	Ordering of wrong test(s)
		E	Test ordered wrong way
			Performance
		F	Sample mixup/mislabeled (eg, wrong patient/test)
		G	Technical errors/poor processing of specimen/test
		H	Erroneous lab/radiology reading of test
		I	Failed/delayed reporting of result to clinician
			Clinician Processing
		J	Failed/delayed follow-up of (abnormal) test result
		K	Error in clinician interpretation of test
5. Assessment			*Hypothesis Generation*
		A	Failure/delay in considering the diagnosis
			Suboptimal Weighing/Prioritization
		B	Too little consideration/weight given to the diagnosis
		C	Too much weight on competing/coexisting diagnosis
			Recognizing Urgency/Complications
		D	Failure/delay to recognize/weigh urgency
		E	Failure/delay to recognize/weigh complication(s)
6. Referral/Consultation		A	Failure/delay in ordering referral
		B	Failure/delay obtaining/scheduling ordered referral
		C	Error in diagnostic consultation performance
		D	Failure/delayed communication/follow-up of consultation
7. Follow-up		A	Failure to refer patient to close/safe setting/monitoring
		B	Failure/delay in timely follow-up/rechecking of patient

© 2022 Schiff GD et al. *JAMA Network Open.*

eTable 2. Reliable Diagnosis Challenges (RDC) Taxonomy

Challenging disease presentation
- Atypical presentation
- Non-specific symptoms and signs
- Unfamiliar/outside specialty
- Findings masking/mimicking another diagnosis
- Red herring misleading findings
- Rapidly progressive course
- Slowly evolving blunting onset perception
- Deceptively benign course

Patient factors
- Language/communication barriers
- Signal:noise -- patients with multiple other symptoms or diagnoses
- Failure to share data (to be forthcoming with symptoms or their severity)
- Failure to follow-up

Testing challenges
- Test not available due geography, access, cost
- Logistical issues in scheduling, performing
- False positive/negative test limitations
- Performance/interpretation failures
- Equivocal results/interpretation
- Test follow-up issues (e.g., tracking pending results)

Stressors
- Time constraints for clinicians and patients
- Discontinuities of care
- Fragmentation of care
- Memory reliance/challenges

Broader challenges
- Recognition of acuity/severity
- Diagnosis of complications
- Recognition of failure to respond to therapy
- Diagnosis of underlying etiologic cause
- Recognizing misdiagnosis occurrence

© 2022 Schiff GD et al. *JAMA Network Open.*

eTable 3. Diagnostic Pitfalls Associated With Neurological Conditions

Correct Diagnosis	Incorrect/Initial Dx	Pitfall; Comments
**Significant, Missed Neurologic Diagnoses**		
cerebellar hemorrhage, infarct	Viral GI illness	Cerebellar infarct missed because nausea and vomiting (even without constipation and/or diarrhea) was dismissed as viral GI illness.
	Other headache	Pt with headache, nausea, vomiting and missed cerebellar stroke until patient becomes somnolent
CVA	vertigo, labyrinthitis	not recognizing stroke symptoms, diagnosing as vertigo or labyrinthitis
	peripheral dizziness	dizziness thought to be peripheral but actually stoke
	peripheral nervous system diseases	foot drop, wrist drop for peripheral -- stroke
	diabetic neuropathy	acute onset limb weakness referred for diabetic neuropathy, diagnosis = stroke
vertebrobasilar disease, TIA, stroke	benign vestibulopathy	dizzy, vestibulopathy when it is vertebrobasilar disease, TIA/stroke
Subdural hematoma	benign headache	headache thought to be benign but was a subdural hematoma
Brain Tumor	Migraine	missing severe headache etiologies and labeling migraines (tumor and temporal arteritis)
	trigeminal neuralgia	confusion with unilateral jaw/face pain confused with trigeminal neuralgia but ultimately malignancy
RCSV, SAH, pseudotumor	benign headache	headache with organic pathology (RCVS, SAH, pseudotumor) treated with fioricet and patient has to present multiple times before imaging
paraneoplastic syndrome	primary psychiatric disease	new onset psychosis attributed to primary psychiatric disease in someone with features of paraneoplastic syndrome
pseudotumor cerebri	migraine	pseudotumor cerebri diagnosed as migraine
Encephalitis	schizophrenia	psychotic break that was assumed to be schizophrenia but was encephalitis
fungal infection	migraine	
amyotrophic lateral sclerosis	weakness	weak limb -> [illegible] without pain -> ALS
	Alzheimer's	dysphagia leading to Alzheimer's work-up when it is ALS
cervical spine lesion		not considering cervical spine lesion as potential cause for leg weakness/gait problem. ordering L-spine MRI and stopping or not ordering c-spine MRI
cervical spondylosis	missed/ overdiagnosed	cervical spondylosis both missed and overdiagnosed (imbalance, hand numbness)
spinal fracture with cord compression	Guillain-Barre syndrome	lower extremity weakness misdiagnosed as Guillain-Barre but was spinal fracture with cord compression
Temporal Arteritis	Migraine	missing severe headache etiologies and labeling migraines (tumor and temporal arteritis)
Guillain-Barre Syndrome	benign paresthesia	Guillain-Barre syndrome subtle tingling dismissed and pt represents when more obvious weakness manifests
autoimmune necrotizing myopathy	toxic myopathy	Rare patients on statins develop pan autoimmune necrotizing myopathy, not typical toxic myopathy.

© 2022 Schiff GD et al. *JAMA Network Open.*

		Patients do not get better with discontinuation of statins but only after treating with immunotherapy
Multiple sclerosis		early signs of multiple sclerosis such as optic neuritis not recognized
optic neuritis	decreased vision	misdiagnosing optic neuritis for decreased vision
parkinsonism	chronic fatigue syndrome	misdiagnosis
Parkinson's Disease		not recognizing parkinsonism, thinking it is weakness, fatigue, tiredness, etc.
Parkinson's Disease	tremor 2/2 shoulder surgery	tremor related to shoulder surgery, diagnosis = Parkinson’s disease
medication induced Parkinson's		missing medication induced Parkinson’s
ulnar neuropathy		not recognizing common entrapment syndromes and doing unnecessary tests/referrals (e.g., ulnar neuropathy)
thoracodorsal radiculopathy	surgical cause abdominal pain	abdominal pain in DM - abdominal w/u [surgery?]
B12 deficiency		not checking methylmalonic acid in low normal b12 levels in patients with symptoms
Medication Overuse Headache	Headache	headache/pain management with medication overuse causing overmedication headaches or causing somnolence with overmedication
**Misdiagnoses/Overdiagnosis of other diagnoses as more serious Neurologic Disease**		
Overdiagnosis CIPD	chronic inflammatory demyelinating polyneuropathy	not warming up limbs before nerve conduction study and mislabeling as CIPD and treating with IUG
Bell's palsy	stroke	mistaking lower motor neuron facial weakness (Bell's Palsy) thinking it is a stroke
No Seizure disorder	Seizure Disorder	overtreating seizures
Non MS	MS	diagnosis of MS based only MRI, non-specific lesion
		MRIs for non-specific systemic symptoms leads to incident [illegible] MRI findings
vasovagal syncope	seizure, stroke	syncopal events (often vasovagal) referred for extensive work-up for seizure, stroke, etc. "answer" often in better history taking
No TIA; nonspecific sx	TIA	making excessive TIA diagnosis
Anemia or Cardiac Arrhythmia	TIA	not looking at CBC and EKG in TIA patients
mouth infection?	giant cell arteritis	confusion with unilateral jaw/face pain presumed to be giant arteritis, treated with steroids which lead to worsening mouth infection and infective endocarditis
Other neuro non CVA dx	ischemia	assumption all subacute/acute changes in neurologic function are ischemic in etiology
Nonspecific dx	Neuro Lyme	labelling chronic symptoms as neuro lyme
functional disorder, conversion disorder	peripheral nervous system diseases	misdiagnosis of peripheral nervous system disease in functional or conversion disorder
	organic disease	misdiagnosis of functional neurological symptom disorder as an organic disease and preforming excessive diagnostic tests and initiating potentially harmful treatments (ex: AED [?anti-epileptic drug])

© 2022 Schiff GD et al. *JAMA Network Open.*

psychiatric symptoms with somatization manifestations	Neurologic condition	referral for psychiatric symptoms with somatic manifestations and no clear clinical symptoms/signs of neurologic disease
neurologic illness	psych illness	new psychiatric symptoms attributed to psych illness rather than a missed neurologic illness (brain tumor, stroke, encephalitis)
psychogenic non-epileptic seizures	epilepsy	psychogenic non-epileptic seizures misdiagnosed as epilepsy, on meds for months/years
hysterics with non-organic findings	neuro disease	Diagnosing hysterics with outrageously non-organic findings as having neurological disease. Possibly not wishing to address the issue
orthostasis, anxiety	More serious neuro dx causing dizziness	referral for dizziness in patient with orthostasis, anxiety, or other non-neurological etiology
Non neuro benign causes syncope dizziness	Syncope/dizziness due to neuro etiology	syncope/dizziness is usually attributed to neurologic causes other than obvious reasons
No PMR	PMR	Response to steroid mistaken for diagnostic support for PMR.
No MS / asymptomatic	MS (abnormalities of MRI)	MRIs performed for unclear diagnostic reasons, some findings, and then asymptomatic patient referred to exclude MS
Other (potentially more serious) neuro dx	transient global amnesia	over-diagnosed
TMJ		confusion with unilateral jaw/face pain leading to referral when actually just TMJ
muscle disease	liver disease	I see many patients with muscle diseases who initially are misdiagnosed as having a liver disease because routine AST and ALT levels are elevated. Many clinicians forget that these should not be referred as "liver function tests." These enzymes are also present in muscle. I see several patients every year who get abdominal CT/US and even liver biopsies before someone checks a serum CK. This should be done before a dangerous and expensive liver work-up
**Generic/Other Neurology Diagnosis Pitfalls**		
neuro conditions	psych illness	not considering new overt psych symptoms as potentially neurological
Non NPH causes of dementia	NPH	overdiagnosis of NPH
dementia	NPH	misdiagnosis of Normal Pressure Hydrocephalus in cases of dementia
dementia		referrals for dementia evals not early enough
metabolic disease		congenital states can be misinterpreted as focal abnormalities and potentially not diagnosed for toxic/metabolic abnormalities
febrile seizures		unnecessary referrals for simple febrile seizures
ataxia	weakness	confusing ataxia with weakness
mild cognitive difficulties		missing mild cognitive difficulties which manifest as multiple organic complaints
bulbar weakness	appetite problem	mistaking poor PO intake for appetite problem instead of bulbar weakness
Serious HA	benign headache	filtering headache syndromes. In the ED we would commonly see patients whose concerning HA was initially minimized. On the other hand, would see non-concerning HA triaged to the ED.

© 2022 Schiff GD et al. *JAMA Network Open.*

benign headache	Serious HA	benign headache
Benign Headache		MRI of the brain is overperformed, particularly in cases of mild headache and lead to a work-up of incidentally found tumors particularly meningiomas
Benign; unrelated, incidentaloma	Meningioma	attributing symptoms to meningioma that is actually asymptomatic
peripheral neuropathy		numb feet in old people - peripheral neuropathy
afferent papillary light defect		pt with optic nerve dysfunction, but diagnosis is missed because eyes get dilated before dx can be made during eye exam
Physiological Anisocoria	Anisocoria due to neurologic issue	anisocoria that is physiological
Neuro etiologies visual impairment	Ophthalmology visual impairment	vision complaints mis-triaged to ophthalmology
CNS vertigo	Peripheral Vertigo	dizziness approach, usually can't make a correct differential diagnosis between CNS vertigo or peripheral vertigo
Non-specified Neuro "decline"		missing course of illness/history because patients are not forthcoming about their decline
Autism		delayed referrals for language delay w/u or autism w/u
neurodegenerative disorder	missed dx	missing diagnosis of neurodegenerative disorder, thereby not giving treatment that can improve patient quality of life
Increasing head circumference in infants		delayed referrals for head ultrasounds for rapidly increasing head circumference in infants
		ordering unnecessary tests (esp. MRI) for psychosomatic complaints
		MRI obtained without clear indication (e.g., dizzy, headache without feature) leading to nonspecific findings, leading to referral and sometimes requiring testing that would otherwise not be necessary

Data source: Derived from 836 relevant cases among 4,325 patient safety incident reports, 403 closed malpractice claims, and 24 M&M reports, and 355 focus groups responses.

© 2022 Schiff GD et al. *JAMA Network Open.*

eTable 4. Diagnostic Pitfalls Associated With Breast Cancer

Disease-specific diagnostic pitfall	No.	Example of cases demonstrating pitfall
1. Family History Issues	4	• Failure to obtain family history of breast cancer<br>• Under-weighing family history of breast cancer
2. Atypical Presentation/ Cognitive Challenges	6	• Underestimating risk of BC in young symptomatic patients<br>• Fast-growing cancers arising during MMG interval<br>• Under-weighing complaints of patients with psychiatric diagnoses<br>• Prioritizing chronic medical or social issues over screenings in complex patients
3. False Negative Physical Exam	2	• Lump felt to be benign on physical exam<br>• Bias in wanting to reassure patient, due to low likelihood of BC
4. Fibrocystic/Dense Breast Dilemmas	9	• Fibrocystic breast tissue can obscure underlying BC in MMG<br>• Not recognizing changes in breast density over time<br>• Failure to investigate unilateral fibrocystic changes<br>• Failure to investigate breast lump with FNA in patient with dense breasts and negative U/S
5. Screening vs. Diagnostic Mammogram Order	2	• Ordering/performing a screening MMG, rather than a diagnostic MMG
6. False Negative Mammogram	9	• False negative MMG in pt with fibrocystic breasts<br>• Failure to reevaluate breast complaints in light of previously negative MMG<br>• Misreading of MMG by radiologists<br>• Failure to follow-up on nipple retraction observed on MMG, attributing it to imaging technique<br>• Falsely reassuring negative “additional views”
7. False Negative Ultrasound	2	• Falsely reassuring negative U/S in pts with breast lump
8. Surgical Referral	4	• Failure to refer to breast surgeon<br>• Breast lump appearing benign to surgeon palpation<br>• Patient failure to follow-up on referral
9. Biopsy Performance/ Interpretation	1	• Inability to recognize missed sampling due to bleeding/complications and failure to repeat biopsy
10. Failure to Order Further Studies	2	• Failure to order diagnostic imaging studies (MMG and U/S)<br>• Failure to recommend excisional biopsy
11. Diffusion of Responsibility/ Coordination Issues	4	• Failure to document/ensure pt was receiving screening MMGS and breast exams<br>• Failed coordination/communication between PCP and GYN
12. Other Symptoms	8	• Failure to follow-up on resolution of mastitis<br>• Failure to pursue etiology of persistent galactorrhea<br>• Pursuing lymphoma as cause of lymphadenopathy<br>• Axillar lymphadenopathy lost due to fact that not incorporated into BIRADS coding (revised now)<br>• Failure to work up persistent painful cyst

Data source: Derived from 836 relevant cases among 4,325 patient safety incident reports, 403 closed malpractice claims, and 24 M&M reports, and 355 focus groups responses.

© 2022 Schiff GD et al. *JAMA Network Open.*

eTable 5. Generic Diagnostic Pitfalls Associated With Most Frequent Conditions

**Diagnostic Pitfalls**	**Overall (N=241)**	**Colon cancer (N=46)**	**Lung cancer (N=43)**	**Prostate cancer (N=34)**	**Breast cancer (N=33)**	**Myocardial infarction (N=29)**	**Sepsis (N=16)**	**Stroke (N=16)**	**Pulmonary Embolism (N=11)**	**Thyroid cancer (N=7)**	**Melanoma (N=6)**
	No. (%)										
Failure to follow-up	68 (28)	10 (22)	14 (33)	16 (47)	14 (42)	6 (21)	-	-	-	4 (57)	4 (67)
Limitations of a test or exam finding not appreciated	62 (26)	5 (11)	12 (28)	8 (24)	14 (42)	12 (41)	-	4 (25)	2 (18)	3 (43)	2 (33)
Disease A repeatedly mistaken for Disease B	58 (24)	9 (20)	11 (26)	5 (15)	-	12 (41)	6 (38)	8 (50)	3 (27)	-	4 (67)
Risk factors not adequately appreciated	39 (16)	10 (22)	-	15 (44)	4 (12)	7 (24)	-	-	3 (27)	-	-
Atypical presentation	37 (15)	-	-	4 (12)	15 (45)	8 (28)	3 (19)	7 (44)	-	-	-
Counter-diagnosis cues overlooked (e.g., red flags)	25 (10)	21 (46)	-	-	-	-	4 (25)	-	-	-	-
Communication failures between primary care physician and specialist	18 (7)	7 (15)	4 (9)	-	4 (12)	3 (10)	-	-	-	-	-
Issues surrounding referral	7 (3)	-	-	3 (9)	-	4 (14)	-	-	-	-	-
Urgency not fully appreciated	6 (2)	-	-	-	-	-	4 (25)	2 (13)	-	-	-
Chronic disease presumed to account for new symptoms	4 (2)	-	-	-	-	-	3 (19)	1 (6)	-	-	-
Miscommunication related to lab ordering	3 (1)	-	-	-	-	-	3 (19)	-	-	-	-
Evolving symptoms not monitored	2 (1)	-	2 (5)	-	-	-	-	-	-	-	-

Data source: Derived from 836 relevant cases among 4,325 patient safety incident reports, 403 closed malpractice claims, and 24 M&M reports, and 355 focus groups responses.

© 2022 Schiff GD et al. *JAMA Network Open.*
